# Optimizing percutaneous reduction and fixation with guidewire modification in pelvic and acetabular fractures: surgical technique and case series

**DOI:** 10.1007/s00590-024-03905-x

**Published:** 2024-03-29

**Authors:** Aiman Mudawi, Osama Alzobi, Jawad Nouraldeen Derbas, Ghalib Ahmed, Maamoun Abousamhadaneh

**Affiliations:** Department of Orthopedic Surgery, Surgical Specialty Center, Hamad Medical Corporation, Doha, Qatar

**Keywords:** Acetabulum fracture, Pelvic ring, Percutaneous fixation

## Abstract

**Background:**

Minimally invasive percutaneous screw fixation for pelvic ring and acetabular fractures has become increasingly popular due to its numerous benefits. However, the precise placement of the screw remains a critical challenge, necessitating a modification of the current techniques. This paper introduces a refined technique employing a modified guidewire to enhance the precision and efficiency of percutaneous fixation in pelvic and acetabular fractures.

**Methods:**

This study details the surgical techniques implemented for correcting guidewire misdirection in percutaneous screw fixation and includes a retrospective analysis of patients treated with this modified approach over a three-year period.

**Results:**

In this study, 25 patients with pelvic ring and acetabular fractures underwent percutaneous screw fixation. The cohort, predominantly male (23 out of 25), had an average age of 38 years. The majority of injuries were due to traffic accidents (18 out of 25). Types of injuries included pelvic ring (6 cases), acetabular fractures (8 cases), and combined injuries (11 cases). Various screw types, including antegrade and retrograde anterior column screws, retrograde posterior column screws, and lateral compression screws, were used, tailored to each case. Over an average follow-up of 18 months, there were no additional procedures or complications, such as neurovascular injury or hardware failure, indicating successful outcomes in all cases.

**Conclusions:**

This study introduces a simple yet effective method to address guidewire misdirection during percutaneous fixation for pelvic and acetabular fractures, offering enhanced precision and potentially better patient outcomes. Further research with a larger patient cohort is required for a more comprehensive understanding of its efficacy compared to traditional methods.

**Level of evidence:**

IV. Therapeutic Study (Surgical technique and Cases-series).

## Introduction

Minimally invasive percutaneous screw fixation represents a critical advancement in orthopedic surgery for managing pelvic ring and acetabular fractures, offering significant advantages over traditional approaches [1]. This method, gaining prominence since its introduction by Routt et al. [2], provides several advantages over traditional open reduction and internal fixation, such as less soft-tissue trauma, reduced intraoperative blood loss, lower infection risks, early pain relief, and the possibility of early weight-bearing ambulation [3, 4].

Despite its benefits, including lessened soft-tissue damage and expedited postoperative recovery, the technique demands high precision due to the complex anatomy of the pelvis and proximity to critical neurovascular structures [5]. Achieving success with percutaneous interventions requires not only an intimate knowledge of pelvic anatomy and skill in radiographic imaging but also the ability to execute screw placement with extreme accuracy [4, 6, 7]. Elmhiregh et al. [8] demonstrated the critical role of careful radiographic assessment in avoiding intra-articular screw placement during acetabular surgery, a recognized complication with significant clinical repercussions. The authors also emphasized the efficacy of utilizing specific radiographic views for the detection of potential joint space violations, thereby underlining the importance of precise imaging in both surgical planning and postoperative evaluations [8]. While the literature provides extensive guidance on the paths and radiographic views necessary for correct screw placement, the task remains highly technical. The narrow osseous corridors challenge even the most skilled surgeons, often necessitating repeated attempts and trajectory adjustments to navigate the guidewire safely [9, 10].

This paper introduces a technique refinement designed to mitigate the challenges of guidewire misdirection. The focus is not only on the experiential reportage, but also on the significant findings derived from employing this technique. We detail how this method minimizes the need for multiple guidewire adjustments, thereby enhancing procedural precision and potentially reducing operative time and patient exposure to radiation. These improvements could have a substantial impact on patient outcomes and the standard of care in the management of pelvic and acetabular fractures.

Our objective is to share a practical solution that addresses a critical technical hurdle in percutaneous fixation, offering a path toward safer and more efficient surgical interventions.

## Methods

A retrospective study was conducted on patients treated with percutaneous screw fixation for pelvic ring and acetabular fractures using a modified guidewire technique between August 2020 and August 2023 at a Level I trauma center. This study received approval from the institutional review board of our institution. The surgical procedures were consistently carried out by the same team of highly skilled surgeons. The classification of the pelvic ring injuries was done following the system proposed by Young and his team [9], while the Letournel and Judet system was employed for the classification of acetabular fractures [10].

### Surgical technique

#### Preoperative considerations

The initial and critical step of this technique involves a thorough review of preoperative imaging. For pelvic ring injuries, our protocol includes a standardized imaging approach, routinely performed in all cases. This approach includes obtaining conventional anteroposterior (AP), inlet, and outlet radiographs of the pelvis. Additionally, iliac oblique and obturator oblique radiographs are obtained for detailed preoperative planning in cases of acetabular fractures. Furthermore, each patient undergoes meticulous computed tomography (CT) scanning with 2-mm slices. This comprehensive imaging is essential for understanding the complete extent of the injury, including fracture location, orientation, displacement patterns, comminution, anatomical variations, and available space for screw placement. These imaging modalities serve as crucial prerequisites for the successful application of the modified guidewire technique presented in this study.

#### Setup considerations

Patients with pelvic ring and acetabular fractures are positioned supine on a radiolucent table. The arms are abducted on an arm-board to avoid any interference with the C-arm during the inlet view. 2D fluoroscopy using a C-arm is utilized to confirm adequate reduction and monitor the safety and accuracy of the initial guide wire passage. Prior to the surgical procedure, the abdomen and the injured lower extremity are prepped and draped to ensure a sterile field. For interventions involving retrograde posterior column screw placement, we ensure that there is adequate access to the distal aspect of the ischial tuberosity and that the hip can be fully flexed to 90° and abducted without interference.

#### Guidewire insertion

**Fig. 1:**
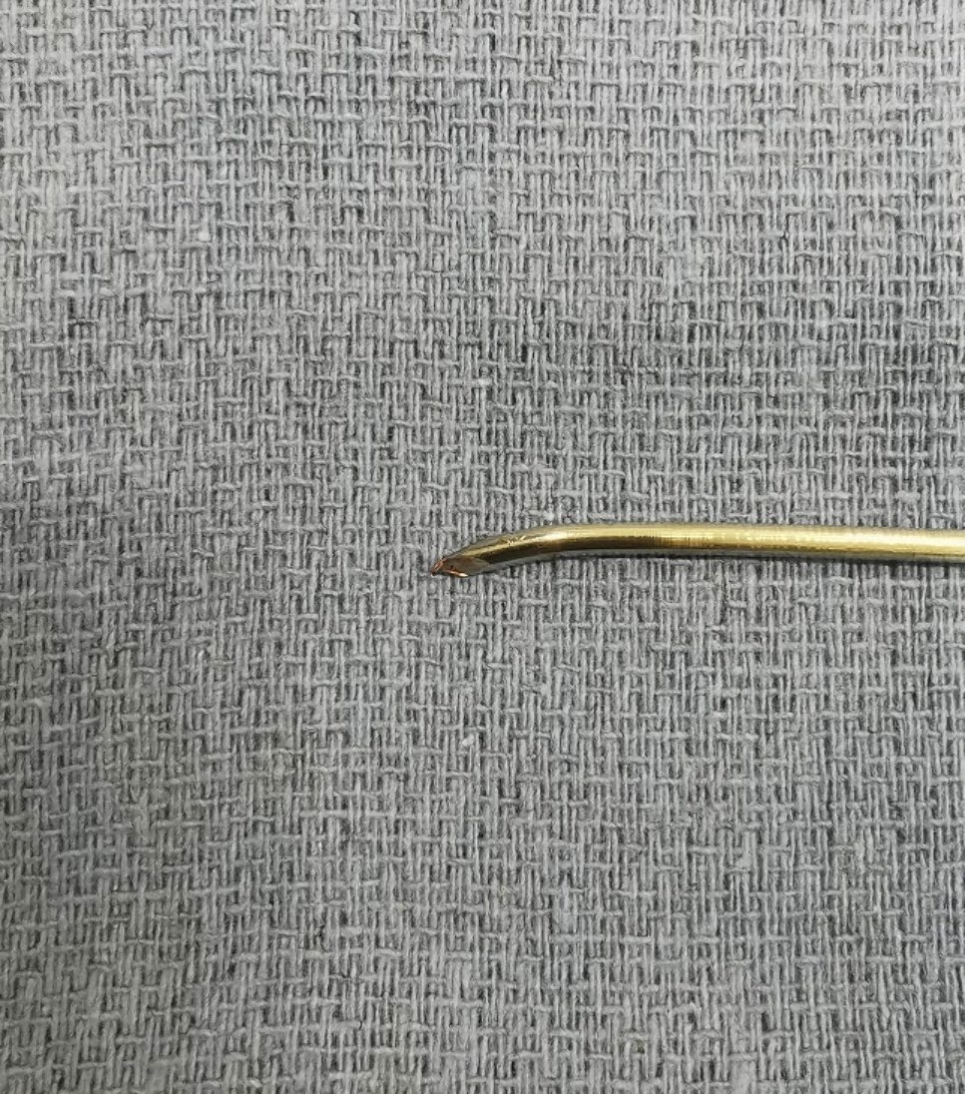
Non-threaded 3.5 mm guidewire with bent tip

**Fig. 2:**
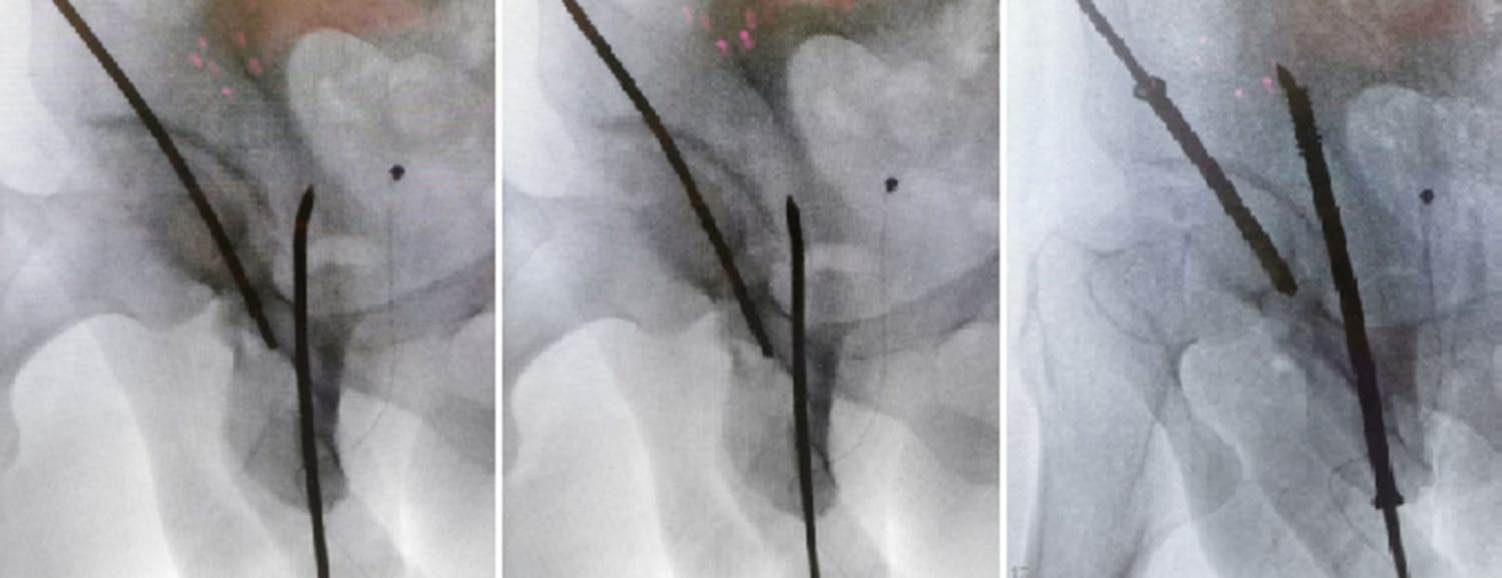
Sequence of intraoperative fluoroscopic pelvic iliac oblique images demonstrating the initial misdirection of the guidewire with bent tip during percutaneous retrograde screw fixation of an acetabular posterior column. The guidewire was carefully retracted and rotated to correct the misaligned trajectory

This technique begins with the standard skin preparation and draping, followed by fluoroscopically identifying the optimal entry point. A 1-cm skin incision is made to expose the entry site, and then a non-threaded 3.5 mm guidewire is used to establish the initial trajectory under fluoroscopic guidance. The sharp end of the guidewire is manually bent, approximately 1 cm from its tip, at an angle of about 20–30° using pliers (Fig. 1). The guidewire is then gently tapped into the bone from the entry point using a hammer. In case of misdirection of the intraosseous trajectory, the guidewire is gently withdrawn and then rotated under biplanar fluoroscopic guidance until the bent tip is oriented in the intended correction direction. A T-handle is utilized for guidewire rotation. Instead of power tools, a mallet is used to delicately insert and advance the guidewire within the bone corridor to ensure superior control without cortical violation. Upon reaching the exit point site with the guidewire, fluoroscopic images are performed to validate the safety of the osseous corridor. If the confirmatory image meets satisfaction, a 4.5-mm cannulated drill is placed over the wire and advanced into the osseous fixation pathway. The screw length is determined using a similar length straight guidewire positioned alongside the modified guidewire at the entry point, with measurements taken extraosseously using a subtraction technique. Once the screw length is determined, a 6.5-mm cannulated screw is used for acetabular column fixation and properly seated with fluoroscopic verification. (Fig. 2). The guidewire is then removed and retrieved through the same skin incision.

#### Percutaneous fracture reduction

**Fig. 3:**
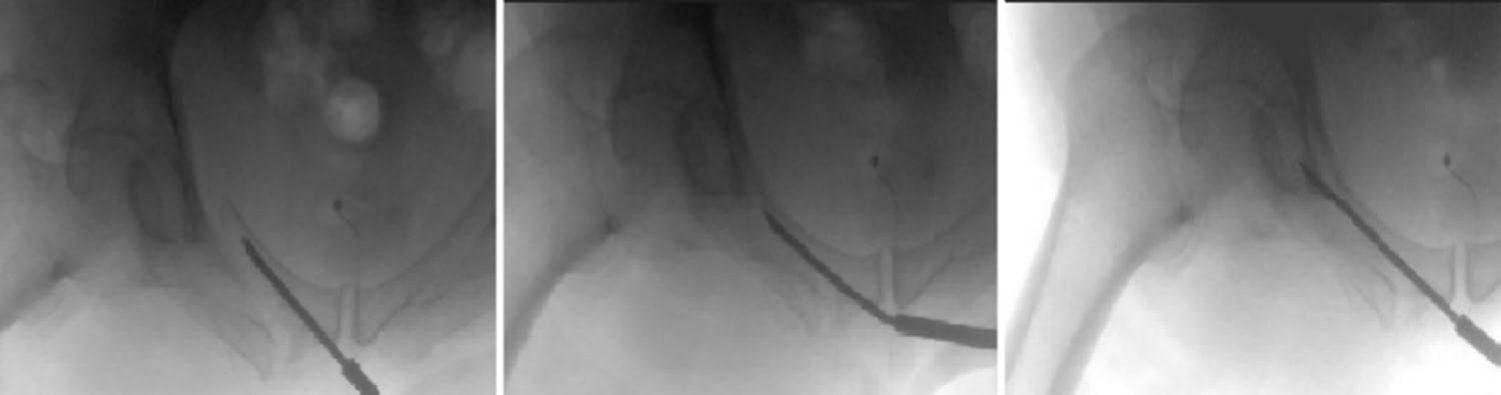
Sequence of intraoperative fluoroscopic pelvic inlet images demonstrating the intramedullary insertion of a 3.5 mm guidewire into the superior pubic ramus. The guidewire was advanced carefully toward the fracture site, followed by the insertion of a protective cannulated drill sleeve over the guidewire to aid in manual manipulation of the fracture

The technique is also effectively utilized in cases of mild displacement or translation at the fracture site. The guidewire with a bent tip is carefully advanced closer to the fracture site and utilized as a joystick to aid in fracture reduction. If more forceful manipulation is required, a 4.5-mm cannulated drill is advanced over the straight section of the guidewire. Subsequently, the cannulated drill is removed, and a protective cannulated drill sleeve is inserted over the guidewire to facilitate manual manipulation of the fracture. Once reduction is achieved, the bent guidewire is advanced beyond the fracture site, engaging the other end under biplanetary fluoroscopic guidance. Correct placement is confirmed using orthogonal fluoroscopic views, and the cannulated drill is advanced until the desired depth is achieved. (Fig. 3).

#### Postoperative care

**Fig. 4:**
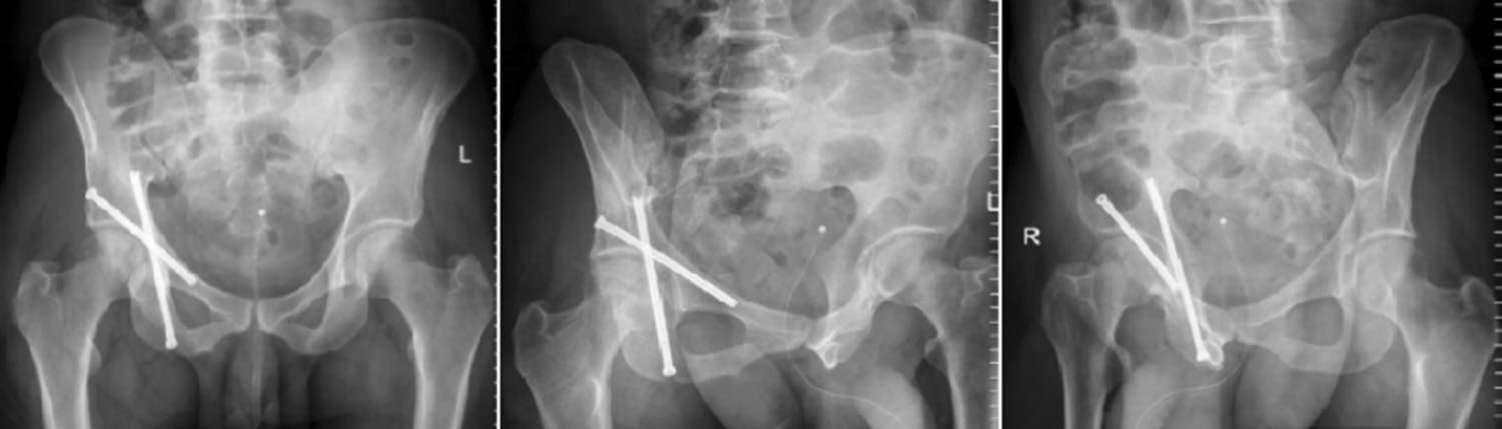
Anteroposterior, obturator and iliac radiographs of the pelvis illustrating the correct alignment of cannulated screws six months after surgery in a patient with a transverse-type right acetabulum fracture. In the radiographs, both columns of the acetabulum are fixed in proper alignment

Postoperative care involves active encouragement for patients to commence early ambulation as part of their recovery process. The timing and manner of weight-bearing, whether using a walker or crutches, are determined based on individual factors such as the fracture pattern and the quality of reduction achieved. Typically, patients remain hospitalized for a duration of 2 to 3 days after the surgical procedure to effectively manage postoperative pain. Postoperative radiographic imaging of the pelvis is routinely conducted to evaluate the final reduction. In select cases, a CT scan may be warranted for further evaluation. (Fig. 4).

## Results

### Patient demographics and injury details

Between August 2020 and August 2023, we identified 25 patients with pelvic ring and acetabular fractures at a Level I trauma center who had complete medical records. The cohort was predominantly male, with 23 males and 2 females. The average age among these patients was 38 years, ranging from 21 to 54 years old. The most common cause of injury was traffic accidents, which accounted for the injuries in 18 patients, while falls were responsible for the injuries in the remaining 7 patients. The distribution of the types of injuries was as follows: 6 patients presented with pelvic ring injuries, 8 with acetabular fractures, and the remaining 11 had a combination of both types of injuries. A summary of the fracture types is provided in Table 1.

**Table 1:** Summary of the injuries

Pelvic ring	Percutaneous fixation only	Open + percutaneous fixation
LC-1	2	
LC-II		1
LC-III	2	1
Acetabulum		
Transverse	2	1
Transverse posterior wall		1
T-shape	1	2
ACPHT		1
Combined	3	8

*LC *Lateral compression*, ACPHT *Anterior column posterior-hemitransverse

### Treatment details

The treatment involved the use of various types of screws, which included anterior column screws (both antegrade and retrograde), retrograde posterior column screws, and LC2 screws (both antegrade and retrograde). It is worth noting that in certain cases, it was necessary to utilize a combination of different screw fixations (Table 2).

**Table 2:** Different types of percutaneous screws placement in patients with pelvic ring fracture and or acetabular fracture

Antegrade anterior column screw	7
Retrograde anterior column screw	6
Retrograde posterior column screw	6
Superior pubic ramus screw	7
LC2 screw	1

### Outcomes

All the patients were subjected to regular follow-ups, with the average follow-up period being 18 months, ranging from a minimum of 3 months to a maximum of 36 months. At the time of the final follow-up, it was observed that none of the patients had the need for any additional procedures or revision surgery. Furthermore, there were no reported complications, such as neurovascular injury, wound infections, loss of initial reduction, nonunion, or hardware failure. This suggests that the treatment was successful and well-tolerated by all patients.

## Discussion

Percutaneous fixation for pelvic and acetabular fractures is a critical tool for trauma surgeons, particularly for managing minimally displaced injuries [11]. Our study has shown that utilizing a modified guidewire technique significantly enhances the precision required for screw placement in these procedures [7]. The results revealed a perfect technical success rate without the need for postoperative interventions or occurrence of complications, suggesting a promising improvement in the standard percutaneous fixation protocol [12, 13].

Traditional methods for percutaneous reduction and fixation in pelvic and acetabular fractures primarily use standard guidewires [7, 11, 12]. While these are fundamentally effective, they occasionally encounter difficulties in unique fracture patterns and anatomical contexts [4]. Our technique, which employs a guidewire with a bent tip, skillfully addresses such misdirections. This not only provides a streamlined and invaluable method for critical corrections, saving vital operative time during percutaneous screw fixation but also facilitates medullary manipulation. This key feature allows for the realignment of displaced pelvic superior ramus and acetabulum column fractures before screw insertion. This modification provides enhanced tactile feedback, making it an alternative choice for surgeons seeking optimized real-time adjustments. While the existing techniques, which sometimes require multiple iterations and potentially heighten the risk of soft tissue damage, our described technique demonstrates a potential advantage in efficiency and precision [7]

The minimally invasive nature of this technique also implies smaller incisions, which generally results in less postoperative pain, reduced scarring, and quicker healing. Alzobi et al. [13] similarly endorsed percutaneous sacroiliac screw fixation, deeming it a safe surgical technique for unstable pelvic trauma, with low complication rates, although they reported a complication related to screw malposition at a rate of 6%. These findings align with the existing literature. Parker et al. in their study of acetabular column fixation, noted that blood loss was under 100 mL for each patient treated with percutaneous fixation [6]. Additionally, Bozzio et al. [14] reported a reduction in fracture displacement with percutaneous surgical treatment and observed good-to-excellent outcomes in patients.

The success of the outcomes greatly depends on precise patient selection. Tailoring the current technique to the fracture type, location, and severity, has been instrumental in maximizing its benefits over traditional open surgeries [15, 16]. This method effectively addressed pelvic ring fractures, notably lateral compression types (I, II, and III). Similarly, for acetabular fractures, it encompassed types from anterior or posterior column transverse and T-shape to combined varieties. While ideal for minimally displaced fractures, more complex cases sometimes required a combined approach of open and percutaneous techniques.

Despite its benefits, the technique presents certain challenges, particularly during the removal of the guidewire after insertion of the cannulated screw. The primary issue arises from the bent-tip part of the guidewire. To address this challenge, several options can be considered. Advancing the guidewire and removing it from the opposite site may be a good solution, but it is only useful in cases of retrograde pubic ramus screw placement. Another strategy involves avoiding hyper-bending of the guidewire tip during insertion. Our recommendation is to limit the degree of bending to 25–30 degrees, thus minimizing the risk of entanglement during removal. Alternatively, the use of a smaller caliber guidewire, such as size 2 or 2.5 mm, for 6.5 cannulated screws can mitigate this issue. However, it is important to note that a smaller guidewire may not serve as an effective reduction tool to correct minor displacements at the fracture site. Following cannulated drilling with a 4.5-mm drill over the guidewire, replacing the bent-tip guidewire with a similar or smaller size straight guidewire is another potential solution.

The main limitation of this technique lies in its infrequent necessity. It is typically required in specific cases, making its application less common. However, surgeons who are familiar with percutaneous fixation of pelvic and acetabular fractures can apply this technique with a relatively small learning curve. Moreover, it is important to note that not all acetabular fracture types are suitable for percutaneous fixation. At our institution, when faced with fractures unsuitable for this approach, we revert to the standard open techniques, using well-established extensile approaches. Weaknesses of this study include its retrospective nature. Additionally, standardized functional outcomes were not systematically collected during routine follow-up appointments with the patients. It is important to note that the purpose of this manuscript was to report on a practical and simple solution addressing the challenges of guidewire misdirection, thereby minimizing the need for multiple guidewire adjustments in patients with narrow and challenging osseous corridors in the pelvic ring or acetabulum. Our focus was not on evaluating the clinical or functional outcomes of a diverse group of pelvic ring injuries and acetabular fractures.

In summary, the utilization of a modified guidewire with a bent tip offers a versatile solution for trauma surgeons in enhancing the precision of percutaneous screw placement within pelvic and acetabular fractures. This technique not only facilitates minor yet crucial corrections to the initially misaligned screw trajectory but also proven effective in addressing specific fracture malalignments prior to percutaneous screw fixation. However, additional research involving a larger cohort is essential to conclusively ascertain the effectiveness of this construct compared to established alternatives.

## Data Availability

All date will be available on request.
